# Fibrous Tumor of the Breast: Case Report of an Underrecognized Entity

**DOI:** 10.4061/2010/847594

**Published:** 2011-01-12

**Authors:** Nilotpal Chowdhury, Ramachandra V. Bhat, Partho Protim Barman

**Affiliations:** Department of Pathology, Aarupadai Veedu Medical College, Bahour Commune Panchayat, Pondicherry 607402, India

## Abstract

Fibrous tumor of the breast is an underappreciated, distinctive, benign, nonrecurrent lesion of the breast. The cytological features of this condition are not well characterized. We present a case report of a 30-year-old female presenting with a hard mass in her right breast. Fine needle aspiration showed smears of low cellularity showing a few clusters and sheets of mostly uniform benign epithelial cells, some of which were lined by myoepithelial cells. Scattered bipolar bare nuclei or stromal fragments were not seen. Excision with subsequent histopathological examination revealed a well-circumscribed, heavily collagenous tumor with atrophy and replacement of the epithelial and ductal elements of the breast, and diagnosed as fibrous tumor. Being nonrecurrent, it is important to distinguish this lesion from fibromatosis of the breast.

## 1. Introduction

Fibrous tumor of the breast is a distinct disease entity characterized by a discrete breast mass composed of collagenized breast stroma along with hypoplasia of ductal and epithelial elements. This condition is comparatively rare and underrecognized. We present a case of fibrous tumor of the breast with the fine needle and histopathological features and discuss its clinical significance and differential diagnosis.

## 2. Case Report

**Figure 1:**
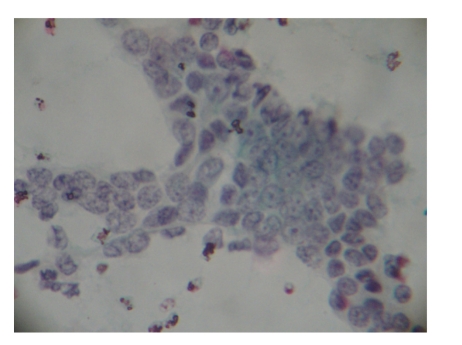
Smears prepared from fine needle aspirates show occasional sheets of benign ductal epithelial cells (Pap, ×400).

**Figure 2:**
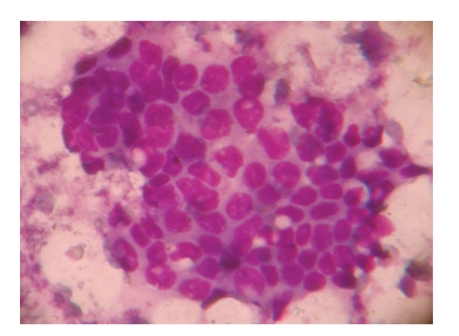
Giemsa-stained smear prepared from fine needle aspirate shows sheet of benign ductal cells with lining myoepithelial cells.

A 30-year-old female presented with a mass on the upper outer quadrant of the right breast with a hard mass measuring 6 cm in diameter. Fine needle aspiration revealed smears of scant cellularity showing sheets of benign epithelial cells with some cells showing mild atypia. There was no stromal component, nor were there any benign bipolar cells in the background (Figures 1 and 2).

**Figure 3:**
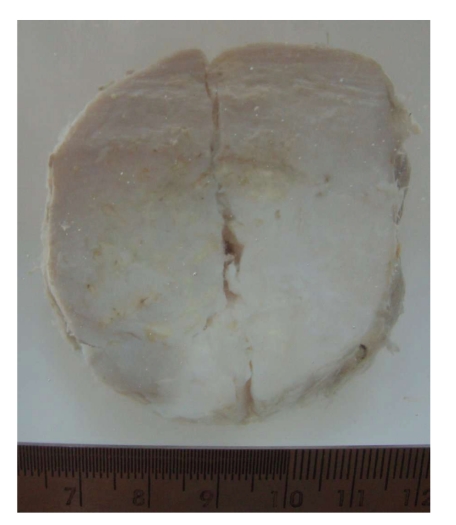
Excised specimen showing well-circumscribed tumor with solid fibrous cut surface and some calcific specks. The superficial cut along the center was made in the surgery to have a closer look.

Excision showed a well-circumscribed mass measuring 5 cm in diameter, with a solid, fibrous cut surface, and calcific specks (Figure 3). 

**Figure 4:**
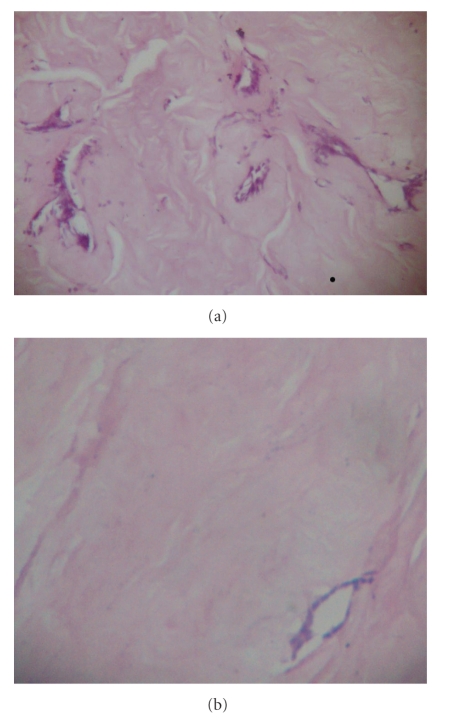
Histopathologic sections show a heavily collagenized stroma with occasional atrophic epithelial elements.

Histologically, the tumor comprised of predominantly densely collagenized stroma, with atrophic ductal and epithelial elements (Figures 4(a) and 4(b)). There was absence of vascular or pseudovascular spaces, nerves, or any lymphocytic infiltrate. Based on these features, a diagnosis of fibrous tumor of the breast was given. However, there were large areas of calcification, which is not a recognized feature of fibrous tumor of the breast.

## 3. Discussion

This tumor has been given various names, ranging from fibrous mastopathy to fibrous tumor and focal fibrous disease. This disease was first characterized by Haagensen [1], who characterized this disease as a distinct clinicopathological entity to be differentiated from fibrocystic disease. Minkowitz et al. used the term fibrous mastopathy [2] and further delineated the disease as occuring in three progressive types: Type I—comprising of mature acini involved by scant collagenized stroma in a concentric pattern, Type II—characterized by decreased acinar tissue, with coarse collagen bundles dissecting the epithelial elements, and Type III—characterized by an almost complete disappearance of the epithelial elements, with a few remnant ductules surrounded by densely collagenized stroma. Puente and Potel used the term fibrous tumor [3], while Rivera-Pomar et al. used the term focal fibrous disease of the breast [4]. The last named authors also followed a tripartite classification scheme analogous to Minkowitz et al. However, they observed lymphatic channels in type III disease, which leads to the suspicion that many of the entities they were describing were actually pseudoangiomatous stromal hyperplasia, which were not characterized at the time of publication of that study. However, this entity is likely to be underrecognized, as pointed out by Puente and Potel [3] as well as Haagensen [1]. This underrecognition is perhaps responsible for a paucity of the literature on this topic and the absence of this condition in the WHO classification of tumors and tumor-like conditions of the breast.

The main differential diagnoses of this entity are diabetic mastopathy, fibromatosis, hamartoma, fibroadenoma, fibrocystic change, and pseudoangiomatous stromal hyperplasia. Absence of a history of diabetes and lymphocytic infiltrate distinguished this disease from diabetic and lymphocytic mastopathy. The borders were well circumscribed, differentiating it from fibromatosis. There was a paucity of lobules and epithelial elements, ruling out fibroadenoma and hamartoma. Hamartoma was further ruled out by the absence of adipose tissue in the lesion. There was no cystic change, nor any apocrine cells, differentiating it from fibrocystic change. The absence of anastomosing pseudovascular-like spaces went against a diagnosis of pseudoangiomatous stromal hyperplasia. Our case is slightly unusual in that calcification was found in the lesion. We could not find a cause for this calcification. The disease is hypothesized to be due to estrogen exposure, as has been emphasized by a case report showing doubling of the tumor size with increased dosage of estrogen in a postmenopausal woman [5]. This disease is predominantly found in premenopausal women in the third and fourth decades of life and has an entirely benign, nonrecurrent course [1–3]. Therefore it is important for this disease to be differentiated from fibromatosis, which has a higher incidence of local recurrence.

The present case report is also, to the best of our knowledge, only the second case report giving the fine needle aspiration features of this lesion. Our findings of a hypocellular aspirate comprising of a few benign epithelial sheets agree with the previously published study [5]. However, we did not give a definitive benign diagnosis due to the absence of benign bipolar cells in the background. The cytological features are nonspecific and only serve to emphasize the benign nature of the lesion. The collagen is too firm and the stroma too hypocellular for there to be a significant stromal component in the smears prepared from the aspirate.

To conclude, fibrous tumor of the breast is a distinctive, nonrecurrent, underrecognized lesion that has a variety of differential diagnoses. It is particularly important to differentiate this condition from fibromatosis due to the different clinical course. Fine needle aspiration cytology is nonspecific but helps to exclude any epithelial malignancy, and the final diagnosis has to be rendered by histopathology.
